# Curcumin and derivatives function through protein phosphatase 2A and presenilin orthologues in *Dictyostelium discoideum*

**DOI:** 10.1242/dmm.032375

**Published:** 2018-01-01

**Authors:** Marco Cocorocchio, Amy J. Baldwin, Balint Stewart, Lou Kim, Adrian J. Harwood, Christopher R. L. Thompson, Paul L. R. Andrews, Robin S. B. Williams

**Affiliations:** 1 Centre of Biomedical Sciences, School of Biological Sciences, Royal Holloway University of London, Egham, TW20 0EX UK; 2 Neuroscience and Mental Health Research Institute, Cardiff University, CF24 4HQ, UK; 3 Department of Genetics, Evolution and Environment, University College London, Darwin Building, Gower Street, London WC1E 6BT, UK; 4 Department of Biological Sciences, Florida International University, Miami, Florida International University, Miami, FL 33199, USA; 5 Division of Biomedical Science, St George's University of London, SW17 0RE, UK

**Keywords:** *Dictyostelium discoideum*, Curcumin, Presenilin, PP2A, Cancer, Alzheimer's disease

## Abstract

Natural compounds often have complex molecular structures and unknown molecular targets. These characteristics make them difficult to analyse using a classical pharmacological approach. Curcumin, the main curcuminoid of turmeric, is a complex molecule possessing wide-ranging biological activities, cellular mechanisms and roles in potential therapeutic treatment, including Alzheimer's disease and cancer. Here, we investigate the physiological effects and molecular targets of curcumin in *Dictyostelium discoideum*. We show that curcumin exerts acute effects on cell behaviour, reduces cell growth and slows multicellular development. We employed a range of structurally related compounds to show the distinct role of different structural groups in curcumin's effects on cell behaviour, growth and development, highlighting active moieties in cell function, and showing that these cellular effects are unrelated to the well-known antioxidant activity of curcumin. Molecular mechanisms underlying the effect of curcumin and one synthetic analogue (EF24) were then investigated to identify a curcumin-resistant mutant lacking the protein phosphatase 2A regulatory subunit (PsrA) and an EF24-resistant mutant lacking the presenilin 1 orthologue (PsenB). Using *in silico* docking analysis, we then showed that curcumin might function through direct binding to a key regulatory region of PsrA. These findings reveal novel cellular and molecular mechanisms for the function of curcumin and related compounds.

## Abstract (precis)

**Summary:** To unlock the therapeutic potential of curcumin and related compounds, we employ a tractable model system to characterise their cellular and molecular effects and propose novel targets implicated in disease.

## INTRODUCTION

Natural products obtained from plants have been used for thousands of years as medicines (Butler, 2004; Newman and Cragg, 2007; Gurib-Fakim, 2006). However, the active compound(s) often have complicated pharmacology, with multiple cellular targets and effects, making traditional pharmacological approaches insufficient to understand their biological activity. These factors preclude standard approaches to investigate mechanism(s) of action.

Curcumin (diferuloylmethane) is a flavonoid derived from turmeric, and provides a good example of a natural product with potential therapeutic activity (Aggarwal and Harikumar, 2009; Ghosh et al., 2015). Currently, ∼120 clinical trials have sought to demonstrate its efficacy in the treatment of various diseases (Gupta et al., 2013), yet an analysis published in 2017 reported that only 17 trials have shown positive outcomes (Heger, 2017). To improve our understanding of this compound, it is crucial to identify potential therapeutic targets, and to test related compounds that have improved chemical characteristics (e.g. solubility) (Oliveira et al., 2015) that can focus research on relevant therapeutic outcomes. Curcumin has diverse cellular effects, including the modulation of transcription and growth factors regulating cell growth and cell death, and as an anti- or pro-oxidant (Goel et al., 2008; Prasad et al., 2014; Priyadarsini, 2014; Gupta et al., 2012, 2013; Zhou et al., 2011). Curcumin has also been extensively investigated for the treatment of Alzheimer's disease (AD), Parkinson's disease (PD), multiple sclerosis (MS), cardiovascular diseases, cancer, allergy, asthma, rheumatoid arthritis, diabetes and inflammation (Yang et al., 2017; Lakey-Beitia et al., 2017; Jurenka, 2009; Srinivasan, 1972; Chougala et al., 2012; Zhang et al., 2013; Tang and Taghibiglou, 2017; McClure et al., 2017).

The main limitations of the therapeutic use of curcumin are its poor bioavailability and limited understanding of the cellular effects in relation to its molecular structure (Gupta et al., 2013). Its structure consists of two aromatic rings containing o-methoxy phenolic groups, with a seven-carbon linker consisting of an α,β-unsaturated β-diketone (Priyadarsini, 2014; Ruby et al., 1995; Selvam et al., 1995). Thus, modification of these groups, and analysis of distinct cellular effects and targets, could help with understanding the potential use of curcumin and its derivatives in medicinal roles.

*Dictyostelium* *discoideum* has been used as a tractable model system for the analysis of compounds with potential therapeutic function. It is a eukaryote, with a unique lifecycle including single-celled and multicellular stages, and contains a range of orthologues to disease-linked proteins (Müller-Taubenberger et al., 2013). It has also been used to investigate the molecular actions of structurally and pharmacologically diverse compounds from bitter tastants (Cocorocchio et al., 2015; Robery et al., 2011, 2013), to flavonoids (Waheed et al., 2014), to drugs used in the treatment of bipolar disorder (Williams et al., 1999, 2002) and epilepsy (Chang et al., 2012; Xu et al., 2007; Elphick et al., 2012; Boeckeler et al., 2006). Several of these studies have been successfully translated to *in vitro* and *in vivo* animal models (Chang et al., 2015, 2016; Chang et al., 2013, 2014). In *D. discoideum*, distinct cellular processes, including growth, acute cell behaviour and development provide valuable tools for the analysis of compound function. Numerous studies using *D. discoideum* have employed chemical genetic approaches to identify genes controlling the cellular effects of compounds through screening mutant libraries to identify potential molecular mechanisms of compounds (Williams et al., 1999, 2002; Waheed et al., 2014; Robery et al., 2013). Several recent papers have also developed an approach to monitor the acute effects of compounds by measuring changes in cell behaviour (Cocorocchio et al., 2015; Robery et al., 2011). *D. discoideum* is also widely used as a model to investigate development, where cells during starvation aggregate and differentiate to form multicellular fruiting bodies (Marée and Hogeweg, 2001). Thus, using *D. discoideum* provides an advantageous system to analyse the cellular and molecular effects of complex natural products.

In this study, we employ *D. discoideum* to investigate the cellular and molecular targets of curcumin, for which previous studies have demonstrated sensitivity (Garige and Walters, 2015; Swatson et al., 2017). We initially corroborated the effects of curcumin on cell growth and development and further showed an effect on acute cell behaviour (Garige and Walters, 2015; Swatson et al., 2017). To differentiate these cellular effects and mechanisms, we then employed a range of complex natural and synthetic curcumin derivatives to highlight key functional groups of curcumin and differentiate these effects from antioxidant activity. A chemical genetic approach was then used to investigate the molecular targets of curcumin and a synthetic derivative. From this approach, two proteins were identified – the protein phosphatase 2A regulatory subunit PsrA (Lee et al., 2008), associated with cancer onset (Kiely and Kiely, 2015), and the presenilin 1 orthologue PsenB (Ludtmann et al., 2014), implicated in AD (De Strooper and Annaert, 2010) – which partially control the effects of these compounds. *In silico* modelling of curcumin binding sites was then used to predict a binding site on PsrA.

## RESULTS

### Curcumin shows distinct effects on acute cell behaviour, growth and development in *D. discoideum*

**Fig. 1.**
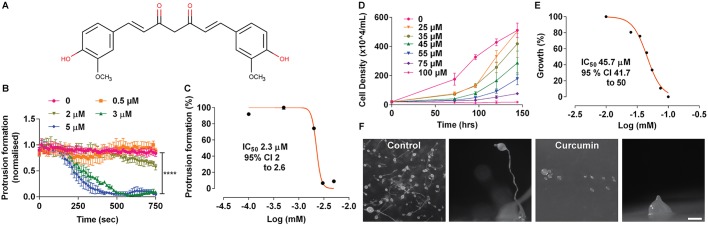
**Acute cell behaviour, growth and developmental effects of curcumin on *D.*** ***discoideum*****.** (A) Curcumin, a diferuloylmethane, was used to assess multiple roles using *D. discoideum* as a model. (B) Time-dependent changes in *D. discoideum* cell behaviour (membrane protrusion) were recorded over a 15-min period (±s.e.m.) at increasing concentrations of curcumin. Data are presented normalised to control conditions, showing a significant difference between control condition (vehicle) and 3 µM (****P<0.0001) using one-way ANOVA. (C) The concentration-dependent response is illustrated as the normalised reduction of cell behaviour (protrusion formation) against the Log (concentration) of curcumin, enabling calculation of an IC_50_ with a 95% CI. (D) *D. discoideum* cells were grown with increasing concentration of curcumin, causing a complete block at 100 µM, with (E) normalised concentration-dependent response shown plotted against Log curcumin (mM) concentration, providing an IC_50_ with 95% CI. (F) Cells were developed on agar over 22 h in control conditions (vehicle) and in the presence of 100 µM curcumin. Scale bar: 0.25 mm for both side view images. All experiments were carried out in triplicate.

To investigate the cellular effects of curcumin on *D. discoideum*, we initially assessed acute cell behaviour changes following compound exposure (Fig. 1). In these experiments, rapid cell movement was induced through starvation in the presence of pulsatile cyclic AMP (cAMP), leading to the expression of a discrete set of genes (Santhanam et al., 2015). Cell behaviour was recorded using time-lapse microscopy for a period of 15 min including pre- (5 min) and post- (10 min) curcumin addition and computer-aided cell tracking was used to analyse changes in membrane protrusions, normalised to average protrusions pretreatment (Fig. 1B). From this analysis, acute cell behaviour was unaffected at concentrations ≤2 µM but showed a concentration-dependent reduction at increasing concentrations, with a complete block at 3 µM (****P<0.0001; Fig. 1B). By plotting average cell behaviour following treatment against curcumin concentration, a nonlinear regression analysis was used to calculate an IC_50_ for the effect of curcumin on acute cell behaviour as 2.3 µM [95% confidence interval (CI) 2.0-2.6 µM] (Fig. 1C). These data show an acute effect of curcumin on cell behaviour in *D. discoideum*, suggesting the presence of rapidly modified target(s) involved in cellular behaviour.

We then examined the effect of curcumin treatment on *D. discoideum* cell growth. In these assays, growth was assessed in the presence of increasing concentrations of curcumin over 7 days at concentrations ranging from 0 to 100 µM (Fig. 1D). By plotting the rate of exponential growth at each concentration, an IC_50_ value was calculated by nonlinear regression curve fitting. Curcumin completely blocked cell growth at 100 µM with an IC_50_ of 45.7 µM (95% CI 41.7-50 µM) (Fig. 1E). These data indicate an effect of curcumin on *D. discoideum* cell growth, suggesting cellular target(s) for the flavonoid involved in this process.

Furthermore, the role of curcumin in regulating multicellular development was also assessed. When *D. discoideum* are starved, this causes cells to aggregate and differentiate to ultimately form multicellular fruiting bodies over 24 h. The resulting fruiting body consists of a spore head, containing dormant spores held above the substratum by dead, vacuolated stalk cells (Williams et al., 2006). In this process, a further subset of proteins, partially distinct from those of growth and early development, are employed to enable development. In these experiments, cells were plated onto nutrient-deficient solid media, in the absence or presence of curcumin, at a concentration shown above to block growth, and fruiting body structure was recorded after 24 h. In the absence of curcumin, a field of fruiting bodies was formed, with individual fruiting bodies consisting of spore heads elevated by stalks (Fig. 1F). In the presence of curcumin (100 µM), cells were able to form a reduced number of aggregates and were unable to develop into mature fruiting bodies (Fig. 1F). This suggests an effect of curcumin on *D. discoideum* late development, regulating cellular target(s) involved in differentiation.

### Identifying active moieties in curcumin responsible for distinct effects on acute cell behaviour, growth and development

**Fig. 2.**
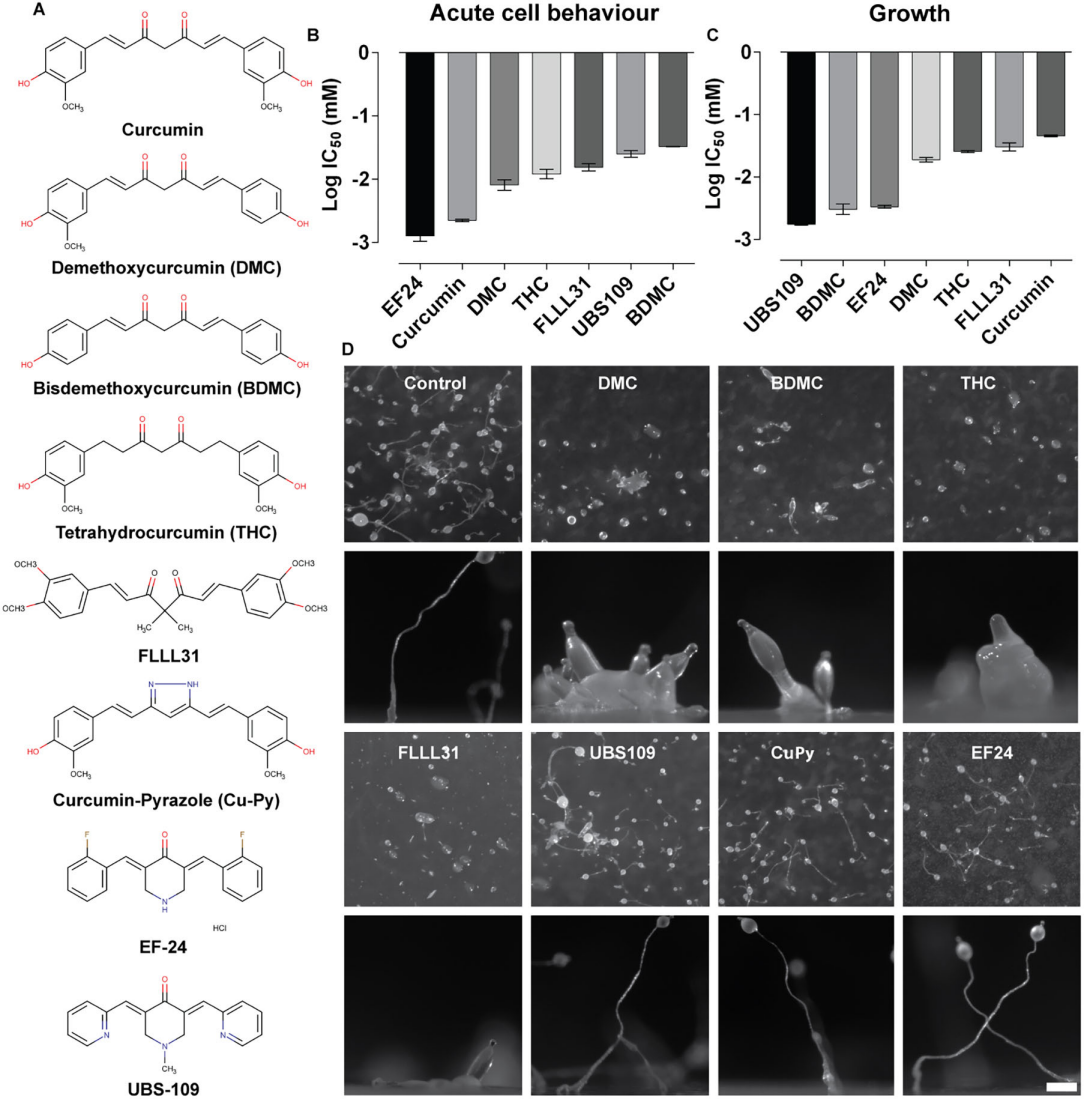
**Quantification of the acute cell behaviour, growth and developmental effects of curcumin derivatives on *D. discoideum*.** (A) Structure of natural and artificial derivatives used in a quantitative structural analysis of curcumin effects in *D. discoideum*. (B) Concentration-dependent responses were determined for cell behaviour (protrusion formation) and illustrated as the IC_50_ for each compound with errors shown as 95% CIs (Figs. S1 and S2). (C) Concentration-dependent responses were determined for cell growth and illustrated as the IC_50_ for each compound with errors shown as 95% CIs (Figs. 3 and 4). Data from B and C are presented as mean±s.e.m. of triplicate experiments. (D) Cells were developed on agar over 22 h in the absence of compounds (vehicle only), or the presence of curcumin derivatives at concentrations shown to block growth (100 µM FLLL31, 25 µM DMC, 20 µM BDMC, 100 µM THC, 6 µM EF24, 5 µM UBS109 and 100 µM CuPy). All images are representative of triplicate experiments. Scale bar: 0.25 mm for all side view images.

To improve our understanding of the distinct effects of curcumin in *D. discoideum*, we employed a range of compounds structurally related to curcumin (both natural and synthetic) to identify structural components of the curcumin molecule that are necessary for distinct effects (Fig. 2A). Analysis of the curcumin-related compounds on acute cell behaviour was carried out as described earlier, with cell behaviour recorded prior to and following the addition of each compound, with data describing a loss of membrane protrusions postaddition (Fig. S1). Secondary plots illustrated dose-dependent effects and provided an IC_50_ value specific to each compound (Fig. S2). From this approach (Fig. 2B), modulation of the phenolic groups through loss of one (demethoxycurcumin; DMC), or both (bisdemethoxycurcumin; BDMC), methoxy groups caused a step-wise reduction in potency in controlling acute cell behaviour (3.5- and 14-fold change, respectively). Similarly, a major metabolite of curcumin, tetrahydrocucrumin (THC), lacking the α,β-unsaturated carbonyl moiety on the seven-carbon linker, leading to loss of the planar structure of the compound, also showed a reduced potency (5-fold change). In addition, loss of the diketone group through formation of the pyrazolic ring (Jadhav et al., 2015) eliminated the effect on acute cell behaviour. Furthermore, FLLL31 (Yuan et al., 2014), which has two hydrogens on the central carbon replaced by methyl groups, and two extra methoxy groups, also showed a reduction in potency (5-fold). Lastly, two structurally distinct compounds, EF24 and UBS109, were investigated, with both compounds considered to be curcumin derivatives (Vilekar et al., 2015; Yamaguchi et al., 2014), where EF24 showed enhanced activity (1.8-fold), and UBS109 showed reduced activity (11-fold), in this model. These data suggest that the diketone moiety is essential for triggering curcumin-dependent inhibition of cell behaviour, and that the presence of the methoxy groups and planar nature of the molecule (lost in THC), and numerous changes to the basic flavonoid chemical composition in the structurally distinct compounds, provided opposite cellular effects on acute cell behaviour.

The assessment of curcumin derivatives on *D. discoideum* cell growth provided insight into chemical moieties of curcumin necessary for this effect. Analysis was carried out as described earlier, with cell growth recorded over 144 h (Fig. S3), and secondary plots illustrating dose-dependent effects and providing IC_50_ values specific to each compound (Fig. S4). For this cellular effect (Fig. 2C), modulation of the phenolic groups through loss of one or both methoxy groups (DMC and BDMC), caused a step-wise increase in potency in controlling growth (2.5- and 14-fold change, respectively). Loss of the planar nature of the compound (THC) also increased potency (1.8-fold change), and the addition of two methyl and methoxy groups in the synthetic analogue FLLL31 increased potency (1.5-fold). Loss of the diketone group through the formation of the pyrazole ring (CuPy) eliminated the effect on acute cell behaviour (and growth). Both structurally distinct compounds, EF24 and UBS109, showed a significant increase (14- and 26-fold change, respectively) in potency in this model. These data suggest that the diketone moiety is essential, and that the phenolic groups play key roles in curcumin activity in regulating cell growth.

Development assays were also carried out to determine which structural components of curcumin control potency in delaying multicellular development in *D. discoideum*. Here, cells were again plated on non-nutrient agar containing curcumin derivatives at concentrations that block growth, and allowed to develop over 24 h, after which fruiting body morphology was recorded (Fig. 2D). In these studies, loss of one or both methoxy groups (DMC and BDMC), loss of the planar structure of the compound (THC), or substitution of the central hydrogens caused delayed and aberrant fruiting body morphology (FLLL31). By contrast, loss of the diketone group through the formation of the pyrazole ring (CuPy) eliminated the developmental effect, and no effect was seen for both divergent structures (UBS109 and EF24). These data suggest that the key moiety of curcumin involved in developmental regulation is the central diketone group.

### Analysis of antioxidant activity of curcumin-related compounds

**Fig. 3.**
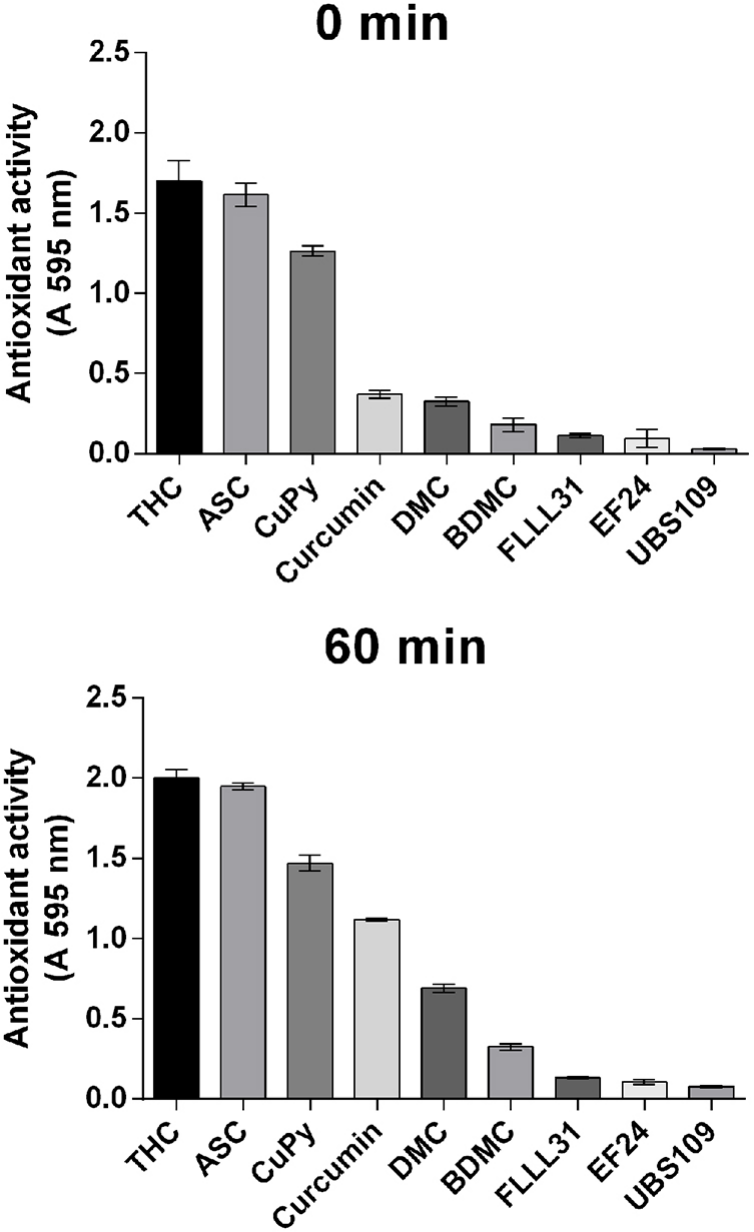
**Antioxidant activity of curcumin and related structures.** Reducing activity was monitored using the FRAP assay. Initial rapid (0 min) and sustained (60 min) activity was measured, using ascorbic acid as an antioxidant standard. Data are presented as mean±s.e.m. of triplicate experiments.

Because curcumin has been widely proposed to function as an antioxidant (Sandur et al., 2007; Gordon et al., 2015), reducing free radicals, we then assessed this activity for curcumin and related compounds. Here, we employed the ferric-reducing ability of plasma (FRAP) assay, monitoring rapid (0 min) and extended (60 min) antioxidant function (Fig. 3). The strong antioxidant, ascorbic acid was used as a control. Of the curcumin-related compounds, THC provided the strongest rapid antioxidant activity, with CuPy also providing an immediate strong effect. Curcumin demonstrated time-dependent antioxidant activity, increasing over the experimental period. Loss of one or two methoxy groups reduced this effect (DMC and BDMC), and the addition of two methoxy groups plus two methyl groups (FLLL31), and modification of the β-diketone moiety or major structural change (EF24 and UBS109), eliminated antioxidant activity (Fig. 3).

### Identification of the molecular targets of curcumin and analogues using a chemical genetic approach

**Fig. 4.**
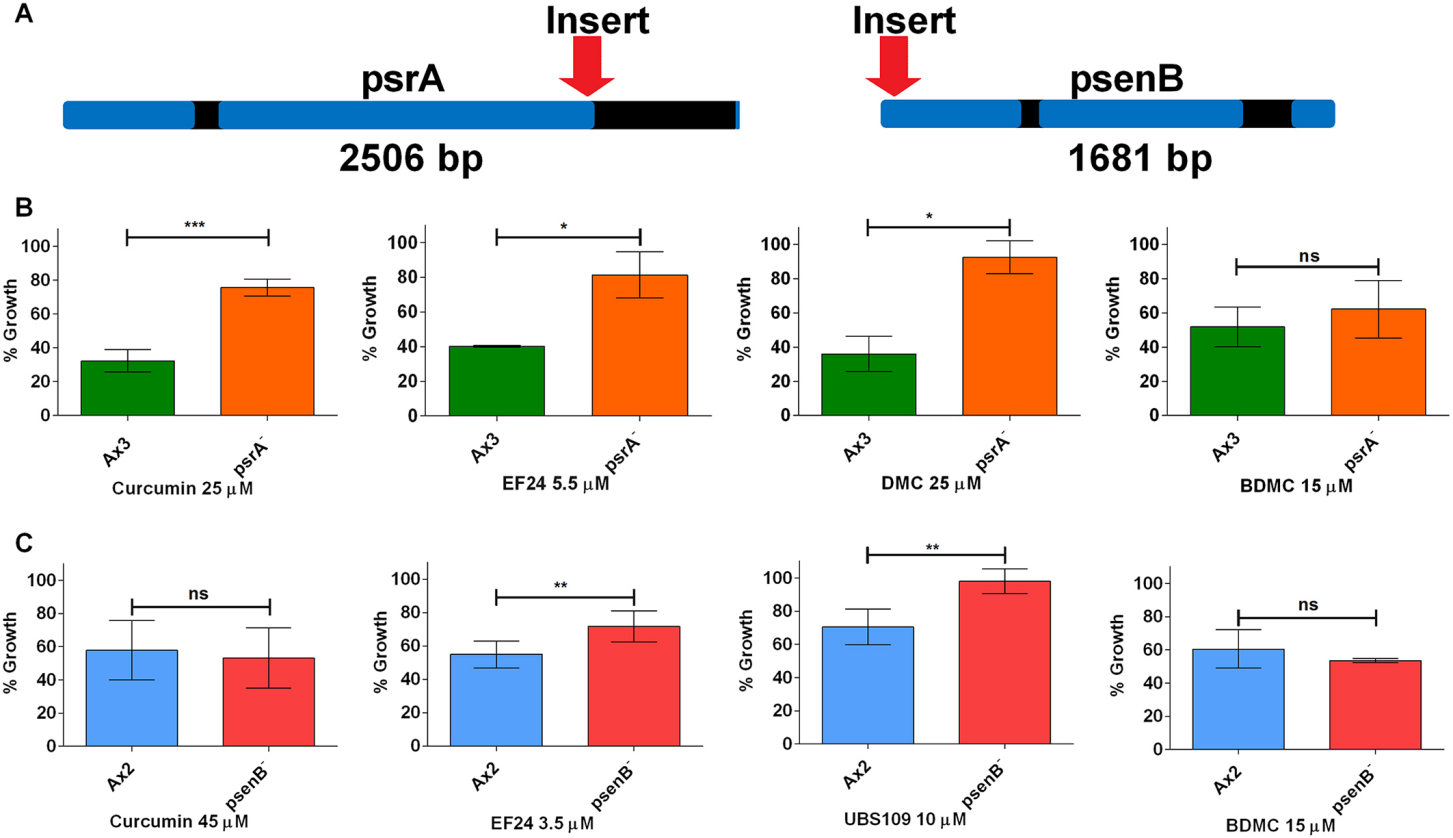
**Loss of *psrA* and *psenB* genes provides partial growth resistance to curcumin or its derivatives.** (A) Through screening a *D. discoideum* mutant library, a mutant showing resistance to curcumin was identified showing an insertion into the protein phosphatase 2A regulatory subunit gene (*psrA*), and a mutant showing resistance to EF24 was identified showing an insertion into the presenilin B gene (*psenB*) (blue exons and black introns). (B) Analysis of wild-type (Ax3) and recapitulated psrA^−^ mutant growth rate confirmed that the psrA^−^ mutant was resistant to curcumin, and additionally to EF24 and DMC, but not BDMC (Fig. S5). (C) Analysis of wild-type (Ax2) and recapitulated psenB^−^ mutant growth rate showed that PsenB was not resistant to curcumin, but showed confirmed resistance to EF24, in addition to UBS109 (Fig. S6). Data are presented as mean±s.e.m. of triplicate experiments. *P<0.05; **P<0.01; ***P<0.001; ns, nonsignificant.

To investigate distinct molecular targets and mechanisms for these compounds, we employed a mutant screen using curcumin and related compounds (Table 1). In these experiments, a library of *D. discoideum* insertional mutants was grown at a concentration of each compound giving an 80-90% reduction in growth over 21 days. Using this approach, a mutant was isolated, showing resistance to curcumin, with the mutagenic cassette inserted into the open reading frame of the protein phosphatase 2A regulatory subunit gene (*psrA*; DDB_G0280469) (Rodriguez Pino et al., 2015) (Fig. 4A). In addition, a mutant was isolated showing resistance to EF24, with the mutagenic cassette inserted immediately downstream of the start codon of presenilin B (DDB_G0292310) (Ludtmann et al., 2014) (Fig. 4A). To confirm that the encoded proteins regulate sensitivity to the compounds, recapitulated mutants were used to assess the rate of exponential growth for each mutant in the presence of the screening compound and a range of related structures over 24 h (Fig. 4B,C; Figs S5 and S6). In the presence of curcumin, psrA^−^ showed significant resistance compared to wild-type cells (*P*<0.001), in addition to resistance to EF24 and DMC (*P*<0.05) (Fig. 4B). Similarly, in the presence of EF24, psenB^−^ showed significant resistance compared to wild-type cells (*P*<0.01), in addition to resistance to UBS109 (*P*<0.01), but not curcumin or BDMC (Fig. 4C).

**Table 1.**
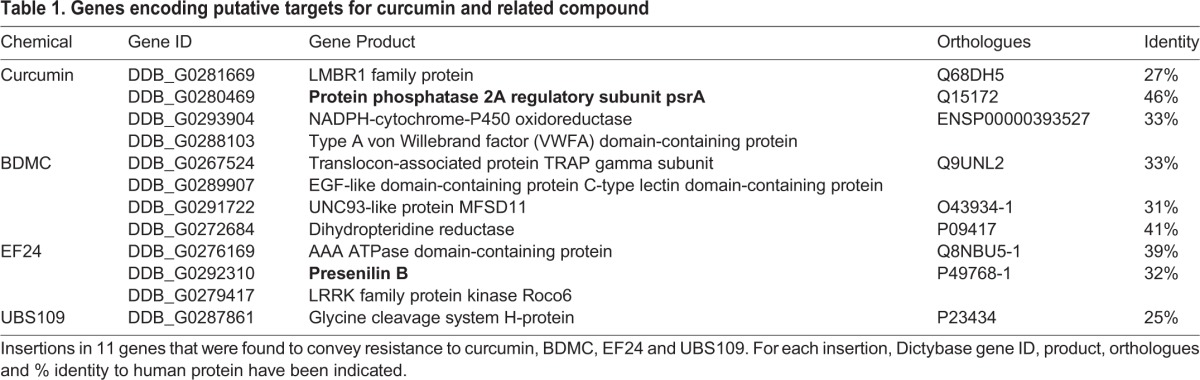
**Genes encoding putative targets for curcumin and related compound**

Because curcumin and EF24 showed effects on both growth and cell behaviour, we also assessed the resistance of psrA^−^ and psenB^−^ to the cell behaviour effects of compounds. In these assays, both mutants were not resistant to curcumin and related compounds (EF24 and DMC) (Fig. S7). These data highlight the distinct mechanisms underlying the effects of curcumin and related compounds on growth and acute cell behaviour.

### Ligand-protein docking prediction of curcumin with PsrA

**Fig. 5.**
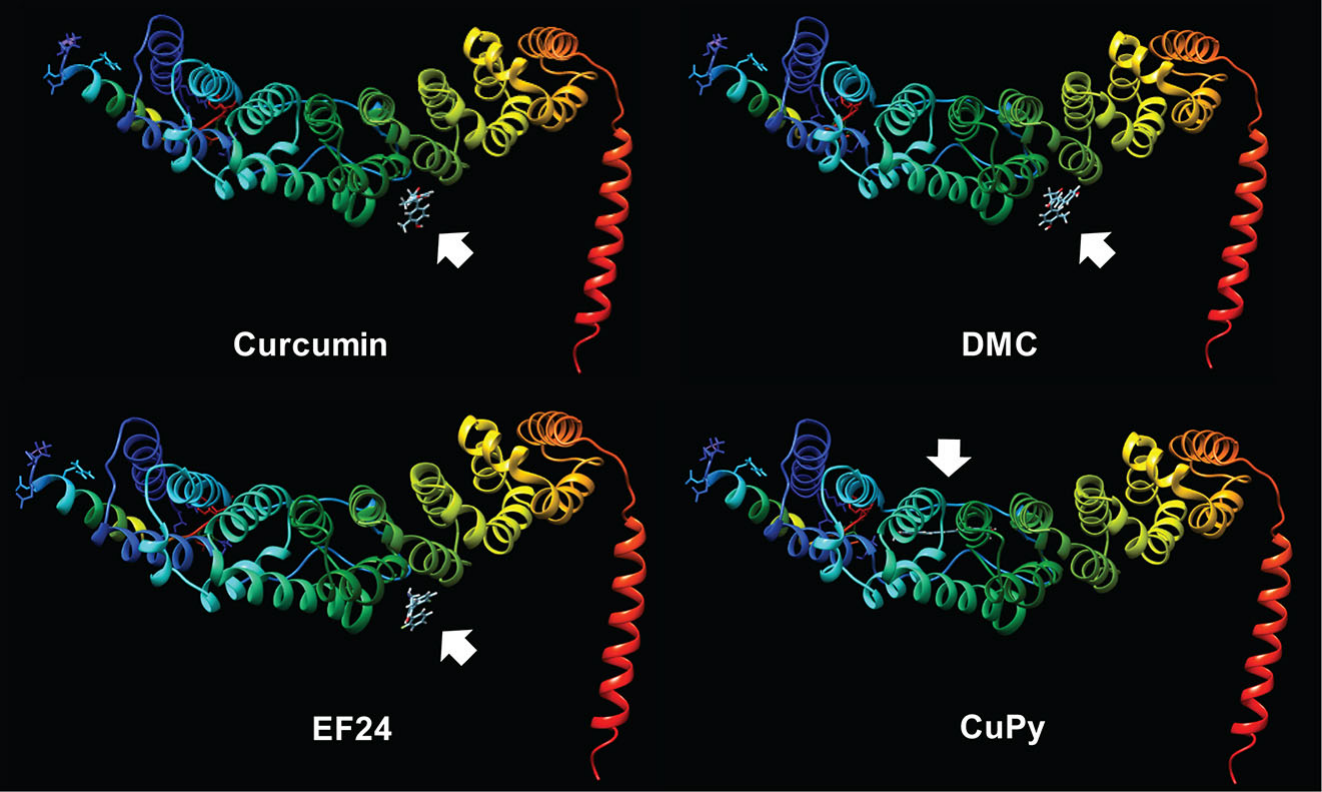
**Molecular docking prediction of PsrA and curcumin analogues.** Tertiary protein structures were generated with Phyre2, with docking prediction performed by SwissDock to provide the most stable binding site (deltaG; Gibbs free energy). Using this approach, curcumin, DMC and EF24 are predicted to bind to the same site on PsrA that is not shown for CuPy.

Molecular docking analysis was used to propose an interaction site for curcumin on PsrA. Here, a tertiary structure of the protein was based upon the mammalian orthologue, PP2A. This tertiary structure was then used to calculate the most stable binding site of curcumin and related compounds, showing the lowest energetic expenditure. This approach identified a common site, on the interface between the regulatory subunit B and the scaffold subunit A (based on crystallography studies of the human PP2A enzyme) (Cho and Xu, 2007) of the protein, which is predicted to bind to curcumin (deltaG −7.39), EF24 (deltaG −7.23) and DMC (deltaG −7.54), but is not targeted by CuPy (deltaG −6.62) (Fig. 5), consistent with the resistance phenotype shown by the psrA^−^ mutant.

## DISCUSSION

Improving our understanding of the potential therapeutic roles of curcumin (Heger, 2017; Gupta et al., 2013) might facilitate its use in medicine. To do this, it is critically important to identify potential therapeutic targets, and to test related compounds that have improved chemical characteristics (e.g. solubility) (Oliveira et al., 2015) that can focus research on relevant therapeutic outcomes. The present study employed a novel system, the social amoeba *D. discoideum*, which enables the dissection of discrete acute, growth and developmental effects of curcumin. Utilising curcumin-related structures identified key chemical moieties responsible for the effects of curcumin, and eliminated an antioxidant mechanism for these effects. The study then identified two novel targets related to disease conditions that might aid in the investigation of its role in therapeutic function.

In this study, we initially quantified the potency of curcumin to regulate distinct aspects of acute cell behaviour, growth and development in *D. discoideum*. We show that curcumin provides the strongest inhibitory effect against acute cell behaviour (with an IC_50_ of 2.3 µM), with reduced potency against cell growth (with an IC_50_ of 44 µM) and development (>100 µM) (Fig. 1). These effects support and extend a previous study (Garige and Walters, 2015), and provide a platform for a comparative study of related chemicals. From these combined data, it is likely that curcumin has more than one molecular target in *D. discoideum* that play distinct cellular roles.

We then adopted a quantitative structure activity relationship approach (QSAR), employing a range of natural and artificial curcumin-related compounds to assess common and distinct cellular effects dependent upon the specific chemistry of the compound (Fig. 2). These studies showed that loss of the diketone group (in CuPy) blocked activity in all three functional roles (acute cell behaviour, growth and development), and modification by substitution of central hydrogens (FLLL31) reduced activity (in acute cell behaviour and growth), highlighting the key role of this group in curcumin function. For the remaining compounds, in acute cell behaviour, curcumin and EF24 showed the most potent activity, with any change in curcumin structure leading to a reduction of activity. By contrast, effects of curcumin-related compounds on growth showed that curcumin is the least potent of all compounds analysed. In development, a common delay was seen for the structures most related to curcumin, with no effect from the divergent artificial analogues (EF24 and UBS109). Independent of the central role of the diketone group, the remaining curcumin-related structures analysed here identified key regions of the chemical structure associated with distinct cell effects. In acute cell behaviour, the most important moieties were represented by the methoxy groups and the planar structure adjacent to the diketone groups, where loss of one or two methoxy groups (DMC and BDMC) strongly and incrementally reduced potency, and loss of the central double bonds (in THC) also reduced potency. However, the two synthetic analogues have a variable potency in blocking cell behaviour. A similar trend is present in growth inhibition, but reversed, where the same molecular substitutions that reduced potency in acute cell behaviour enhanced potency in growth. Interestingly, curcumin and its closely related analogues delayed development, but this was not evident for the synthetic analogues. These data support that curcumin has distinct targets related to acute cell behaviour, growth and development.

Many of the cellular roles for curcumin have been associated with the scavenging of reactive oxygen species (ROS) as an antioxidant. Through the mechanism, antioxidants quench free radicals to inhibit cellular damage (Nimse and Pal, 2015). In this role, specific regions within the curcumin structure (the diketone moiety and two phenolic groups) can undergo oxidation by electron transfer and hydrogen abstraction (Priyadarsini, 2014), and the methoxy groups of curcumin are necessary for antioxidant effects in a range of models (Yang et al., 2017). To investigate whether the effects of curcumin and related compounds on *D. discoideum* were related to this effect, a time-dependent assay was used to assess antioxidant activity (Fig. 6). Surprisingly, THC and CuPy provided the largest rapid-onset activity, with both these compounds and curcumin providing strong activity over an extended period. The remaining compounds showed greatly reduced or no significant antioxidant function, consistent with a crucial role for the diketone and methoxy groups in antioxidant function, but not supporting this effect in the modulation of *D. discoideum* acute cell behaviour, growth or development roles. Similar outcomes for curcumin and its derivatives, shown in anti-inflammatory and antiproliferative effects of human-derived cancer cell lines also did not relate to their ability to modulate ROS (Sandur et al., 2007). Thus, in our study, we have shown that the antioxidant properties of curcumin and its derivatives are not related to the cellular effects in *D. discoideum* observed here, and these effects are therefore likely to occur through other mechanisms.

Studies in animal models or animal-derived cells provide an insight into the potential of natural products as therapies in humans. However, limiting research to these models precludes a range of experiments that could provide important step changes in investigating molecular mechanisms. For example, in *D. discoideum*, a range of novel targets for therapies and natural products have been proposed by using a chemical biology approach (Cocorocchio et al., 2015; Robery et al., 2011, 2013; Waheed et al., 2014; Williams et al., 1999, 2002; Chang et al., 2012; Xu et al., 2007; Elphick et al., 2012; Boeckeler et al., 2006). To apply this approach to curcumin and its derivatives, we identify one gene product, PsrA, regulating the function of curcumin, and a second gene product, presenilin B, regulating the function of the synthetic analogue EF24.

The *D. discoideum* *psrA* gene encodes the orthologue of the mammalian protein phosphatase 2A (PP2A) regulatory subunit B56 (PPP2R5A). In *D. discoideum*, this protein has been shown to regulate cell chemotaxis and differentiation by negatively modulating glycogen synthase kinase 3 (GSK3) protein function (Rodriguez Pino et al., 2015; Lee et al., 2008). PP2A is a major Ser/Thr phosphatase expressed ubiquitously in eukaryotic cells. It is a heterotrimeric enzyme, consisting of a core dimer formed by the scaffolding subunit (A) and a catalytic subunit (C) (Sangodkar et al., 2016). The dimer complexes with one of the several regulatory subunits. In fact, there are >80 different combinations of the PP2A holoenzyme, which regulates the activity and cellular localization of PP2A (Magnusdottir et al., 2009). PP2A regulates a wide variety of cellular processes, including translation, transcription, inflammation, differentiation and apoptosis (Van Hoof and Goris, 2003; Codreanu et al., 2006). PP2A plays a pivotal role in numerous cellular processes, such as cell proliferation, signal transduction and apoptosis, and its deregulation is associated with multiple cancers, AD and increased susceptibility to pathogen infections (Cho and Xu, 2007). It has been shown that in many cancers, there is an abnormal function of the PP2A scaffold and regulatory subunits, supporting its role as a tumour suppressor (Seshacharyulu et al., 2013; Van Hoof and Goris, 2003). In cancer treatment, data are conflicting; patients with a range of cancers improve upon restoring PP2A activity (Kiely and Kiely, 2015), but, conversely, inhibition of PP2A also leads to programmed cell death in many tumour cells. Interest in curcumin as an anticancer treatment is due to a large number of *in vitro* and *in vivo* studies reporting growth arrest of different types of cancer, such as brain, breast, head and neck, liver, pancreas, colon, prostate, ovary and skin cancers (Anand et al., 2008; Kunnumakkara et al., 2008; Ghosh et al., 2015; Klinger and Mittal, 2016; Di Martino et al., 2017; Panda et al., 2017; Singh and Aggarwal, 1995; Bharti et al., 2003). In addition, curcumin has been demonstrated to exert neuroprotective effects by maintaining the levels of PP2A subunit B, leading to tau protein dephosphorylation and/or GSK3β inhibition, which prevents tau hyperphosphorylation (Shah et al., 2015; Sontag and Sontag, 2014; Di Martino et al., 2016).

In our study, we demonstrate that loss of PsrA markedly reduces growth sensitivity to curcumin, EF24 and DMC, suggesting that these compounds might function to regulate cellular activity through this protein. We further propose a potential direct binding of these compounds to PsrA through molecular docking analysis, in which these molecules bind to PsrA but related (inactive) compounds do not. Interestingly, the region of interaction identified in this approach is responsible for interaction with the scaffolding subunit, which might regulate this function. Thus, binding of curcumin and derivatives to the PP2A core regulatory dimer might influence the rate or specificity of binding to the scaffolding subunit and subsequent cellular function. Therefore, this approach has provided a novel insight into a mechanism of curcumin in regulating PP2A activity, with potential impact on therapeutic use.

The *D. discoideum* presenilin B gene encodes one of two presenilin proteins, as part of the γ-secretase complex (Ludtmann et al., 2014). Recent results in *D. discoideum* have shown that presenilin proteins play a conserved noncatalytic role which is independent of proteolytic activity (Otto et al., 2016b). This activity is conserved between human and *D. discoideum* proteins, because the expression of human presenilin 1 in *D. discoideum* restores γ-secretase complex function (Ludtmann et al., 2014). In addition, it has been demonstrated that *D. discoideum* presenilin/γ-secretase activity is required for both phagocytosis and cell-fate determination. Thus, presenilin function and γ-secretase activity are ancient processes that arose prior to metazoan divergence (McMains et al., 2010). The human presenilin 1 (PS1) protein, as a key component of the γ-secretase complex, plays a pivotal role in amyloid precursor protein (APP) cleavage to generate Aβ, where aggregates of Aβ provide a hallmark of AD pathology. In addition, PS1 is a substrate for GSK3β, which is also involved in the pathology of AD (Otto et al., 2016b). Curcumin has been proposed to decrease Aβ production by inhibiting GSK3β-mediated PS1 activation (Zhang et al., 2010; Di Martino et al., 2016), and curcumin downregulates presenilin 1 protein in a dose-dependent manner to regulate γ-secretase function (Yoshida et al., 2011). As a result, curcumin might have neuroprotective effects by inhibiting the generation of Aβ and tau fibrils, but the mechanisms of action remain unknown. In AD, animal models have shown that curcumin reduces amyloid levels and protein oxidisation, which are involved in the cognitive decline process (Baum and Ng, 2004). Furthermore, in patients with AD, macrophages unable to phagocytose Aβ show restored/enhanced activity following curcumin treatment (Zhang et al., 2006). Our study identified and validated a mutant lacking the presenilin B (PS1 homologue) resistant to curcumin-related EF24 and UBS109, and these compounds could provide interesting analogues for further study in the treatment of AD.

Numerous targets and effects have been proposed for curcumin that have led to its investigation for the treatment of several diseases. Targets include transcription and growth factors, cytokines, and regulators of cell growth and death (Goel et al., 2008). Furthermore, curcumin interacts with P-glycoprotein, glutathione, protein kinase C (PKC; PRKC proteins), ATPase, nuclear factor-κB (NFKB1), epidermal growth factor receptor (EGFR), phosphatidylinositol 3 kinase (PI3K; PIK3CA), AKT proteins, mTOR and many other cellular targets (Goel et al., 2008; Prasad et al., 2014; Priyadarsini, 2014; Gupta et al., 2012, 2013; Zhou et al., 2011). In addition, several studies have examined the heptadienedione moiety, which possesses two thiol-reactive α,β-unsaturated carbonyl groups (Fuchs et al., 2009; Ou et al., 2013), that might function to covalently modify cysteine residues of target proteins to regulate cellular functions. These results have stimulated many studies to investigate these targets and effects in a wide range of chronic illnesses such as AD, PD, MS, cardiovascular diseases, cancer, allergy, asthma, rheumatoid arthritis, diabetes and inflammation (Yang et al., 2017; Lakey-Beitia et al., 2017; Jurenka, 2009; Srinivasan, 1972; Chougala et al., 2012; Zhang et al., 2013; Tang and Taghibiglou, 2017; McClure et al., 2017). It remains to be examined if the targets identified in this paper function as upstream modulators or downstream effectors for these curcumin-regulated effects.

In this study, we demonstrated the use of a chemical biology approach to highlight active moieties of curcumin with cellular function using the model system *D. discoideum*. Based upon identified effects, we further employed a chemical genetic approach to identify two possible molecular targets for curcumin and its derivatives, which have been associated with the pathogenesis of cancer and AD in animal models and patients. The study therefore proposes curcumin-related compounds with improved chemical characteristics, which might provide beneficial therapeutic approaches for treating a range of diseases that have been proposed to be curcumin responsive. This approach also highlights a useful model to investigate natural products with multiple cellular effects, and could aid in the development of new therapeutics related to natural products.

## MATERIALS AND METHODS

### Chemicals

The following chemicals were obtained from Sigma-Aldrich (Dorset, UK): curcumin [(E,E)-1,7-bis(4-hydroxy-3-methoxyphenyl)-1,6-heptadiene-3,5-dione; C1386], demethoxycurcumin [(E,E)-1-(4-hydroxy-3-methoxyphenyl)-7-(4-hydroxyphenyl)-1,6-heptadiene-3,5-dione; D7696], bisdemethoxycurcumin [(1E,6E)-1,7-bis(4-hydroxyphenyl)hepta-1,6-diene-3,5-dione; B6938], EF-24 {(3E,5E)-3,5-bis[(2-fluorophenyl)methylene]-4-piperidinone; E8409}, FLLL31 [(E,E)-1,7-bis(3,4-dimethoxyphenyl)-4,4-dimethyl-1,6-heptadiene-3,5-dione; F9057], tetrahydrocurcumin [1,7-bis(4-hydroxy-3-methoxyphenyl)-3,5-heptanedione; 50202], adenosine 3′,5′-cyclic monophosphate (3′,5′-cyclic AMP; A9501, 200 mM stock solution). Curcumin pyrazole {(E)-3,5-bis[β-(4-hydroxy-3-methoxyphenyl)-ethenyl]-1H-pyrazole; SL-318} was obtained from Syninnova. Enaminestore supplied UBS109 [3,5-bis(2-pyridinylmethylidene)-1-methyl-4-piperidone; Z46034963]. All compounds were dissolved in dimethylsulfoxide (DMSO, vehicle).

### *D. discoideum* growth assay

*D. discoideum* cells were harvested and diluted in axenic medium to 2×10^4^ cells/ml. Aliquots of cells (500 µl) were transferred to 24-well plates containing consistent concentrations of solvent (DMSO) in addition to indicated compounds. Cells were grown in 24-well plates, at 22°C, and cell density calculated over 7 days. To provide secondary plots, a rate of exponential growth was calculated (from 72 h to 120 h) at each concentration, and normalised to control (solvent only) conditions.

### *D. discoideum* random cell movement and cell development

*D. discoideum* random cell movement assays and developmental phenotypes assays were carried out as described (Cocorocchio et al., 2015; Robery et al., 2011). In these experiments, behaviour was monitored in cells undergoing random cell movement by taking images every 15 s over a 15-min period, with 5 min recorded prior to, and 10 min after, compound addition. A minimum of three independent experiments for each drug concentration were used, with ≥10 cells quantified per experiment. Protrusions per cell were averaged over the last 5 min of recording, and normalised to control (solvent only) conditions. In these experiments, average (untreated) protrusions per cell were ∼5.7 with a maximum of 6.4 and a minimum of 5.4, consistent with previous reports (Cocorocchio et al., 2015; Otto et al., 2016a).

### *D. discoideum* restriction enzyme-mediated integration screen

To identify mutants in *D. discoideum* resistant to curcumin and analogues*,* two libraries of insertional mutants containing 5000 mutants (psrA^−^) and 11,000 mutants (psenB^−^) were used. Cells were incubated with different concentrations of each compound over 14 days. Mutants growing in the presence of each compound were identified as previously described (Robery et al., 2011; Waheed et al., 2014).

### Mutant growth inhibition assay

Cells were grown in shaking suspensions and harvested in early exponential phase (1.5-2.5×10^6^ cells/ml). Cells were then divided into aliquots and shaken for 24 h in the presence of solvent only or compound, at a concentration which blocked growth by ∼50% in a final volume of 2 ml axenic media. Growth (%) was defined for each wild type and derived mutant (Ax2 and psrA^−^; or Ax3 and psenB^−^), with growth normalised to relevant wild-type cell growth in the absence of compound (solvent only). Each condition tested was carried out at least in triplicate.

### FRAP assay

FRAP solution was prepared by combining 2 ml TPTZ solution (10 mM 2,4,6-tripyridyl-s-triazine in 40 mM HCl), 2 ml FeCl_3_ (10 mM) and 20 ml acetate buffer (300 mM, pH 3.6). The assay was carried out by combining 800 µl FRAP solution with 25 µl of the positive control (1 mM ascorbic acid) or curcumin-related compounds (to give a final concentration of 31.2 µM), and the absorbance was measured at 595 nm. Measurements were obtained in triplicate.

### Protein-ligand docking

Protein sequences were obtained from dictybase.org. The tertiary structure of the *D. discoideum* protein was predicted using Phyre2 (Protein Homology/Analogy Recognition Engine V 2.0) (Kelley et al., 2015). Docking analyses were performed using SwissDock to identify the possible binding sites in PsrA. UFCS Chimera was used to display the results obtained from SwissDock (Grosdidier et al., 2011). Results are expressed as deltaG (Gibbs free energy, where a negative value indicates a spontaneous interaction).

## Supplementary Material

Supplementary information
