# Supplementary material for: Curcumin and derivatives function through protein phosphatase 2A and presenilin orthologues in Dictyostelium discoideum
Source: Dis Model Mech. 2018 Jan 1;11(1):dmm032375. doi: 10.1242/dmm.032375 (PMC5818083; doi:10.1242/dmm.032375)
Supplement: Supplementary information [file dmm-11-032375-s1.pdf]

Supplementary information

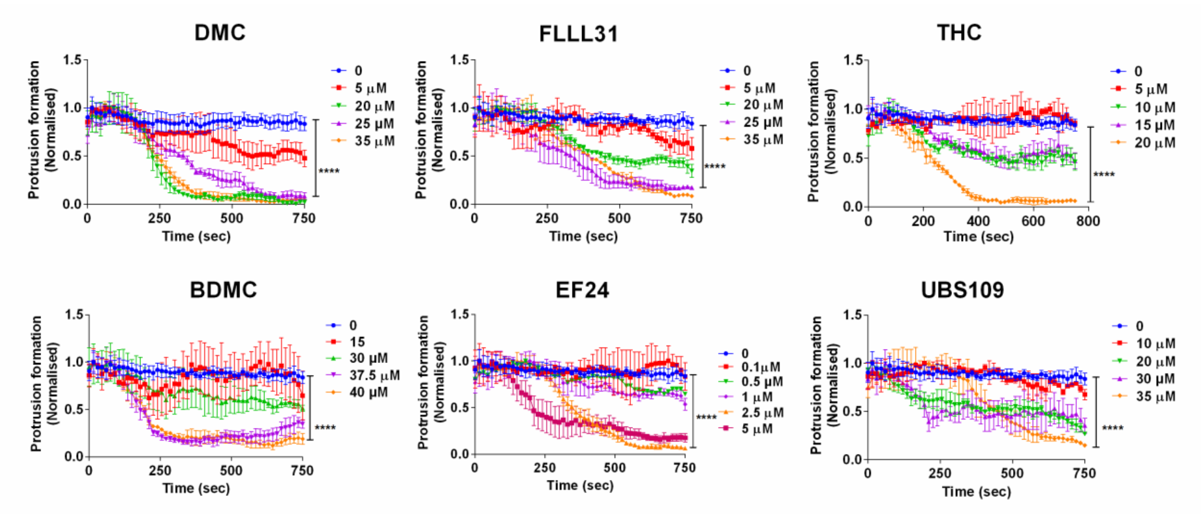

Supplementary Fig. 1. Raw data of *D. discoideum* acute response to curcumin derivatives. Time-dependent changes in *D. discoideum* cell behaviour (membrane protrusion) were recorded over a 15 minute period for triplicate independent experiments (± SEM) at increasing concentrations of six curcumin derivatives to assess their ability to inhibit protrusion formation. The addition of different concentration of each compound at 210 seconds caused a reduction in protrusion formation. Data is presented as normalised to control (vehicle) conditions. Analysis with Two-tailed t-test showed significant changes after the treatment with: DMC 25 µM, FLLL31 25 µM, THC 20 µM, BDMC 40 µM, EF24 2.5 µM and UBS109 35 µM ($p < 0.0001$ ****).

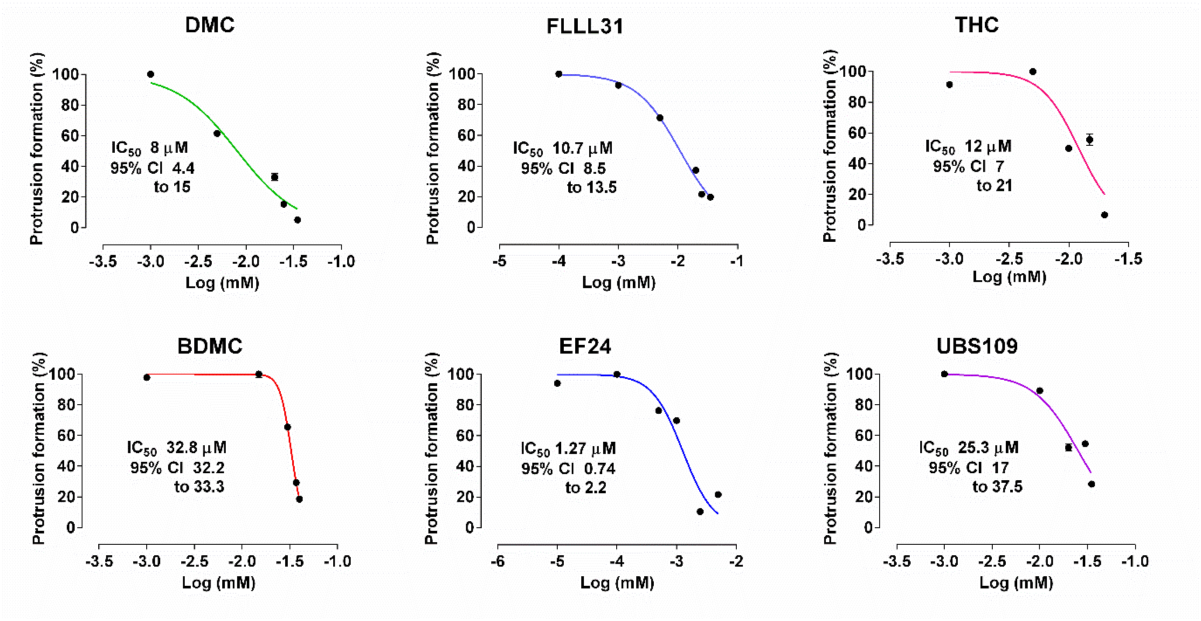

Supplementary Fig. 2. Quantification of the acute effect of curcumin derivatives on *D. discoideum*. Using a range of structurally related compounds, concentration dependent responses were determined for *D. discoideum* cell behaviour (protrusion formation), and illustrated as the normalised reduction in response against the Log (concentration) of each compound (shown with errors based on the 95% confidence intervals), enabling calculation of an IC_{50} values and 95% confidence intervals for each compound.

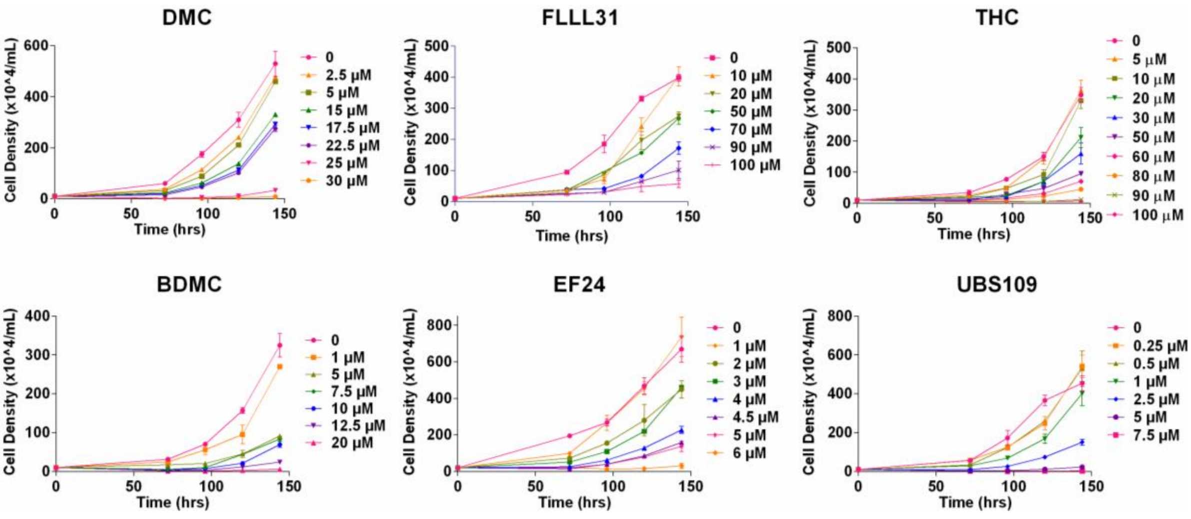

Supplementary Fig. 3. Raw data of *D. discoideum* chronic response to curcumin derivatives. *D. discoideum* cells were grown with increasing concentration of curcumin derivatives in triplicate independent experiments ± SEM. DMC fully blocked growth at 30 µM, FLLL31 and THC at 100 µM,

instead BDMC arrested growth at 20 µ, EF24 and UBS109 inhibiteg proliferation at 6 and 5 µM respectively.

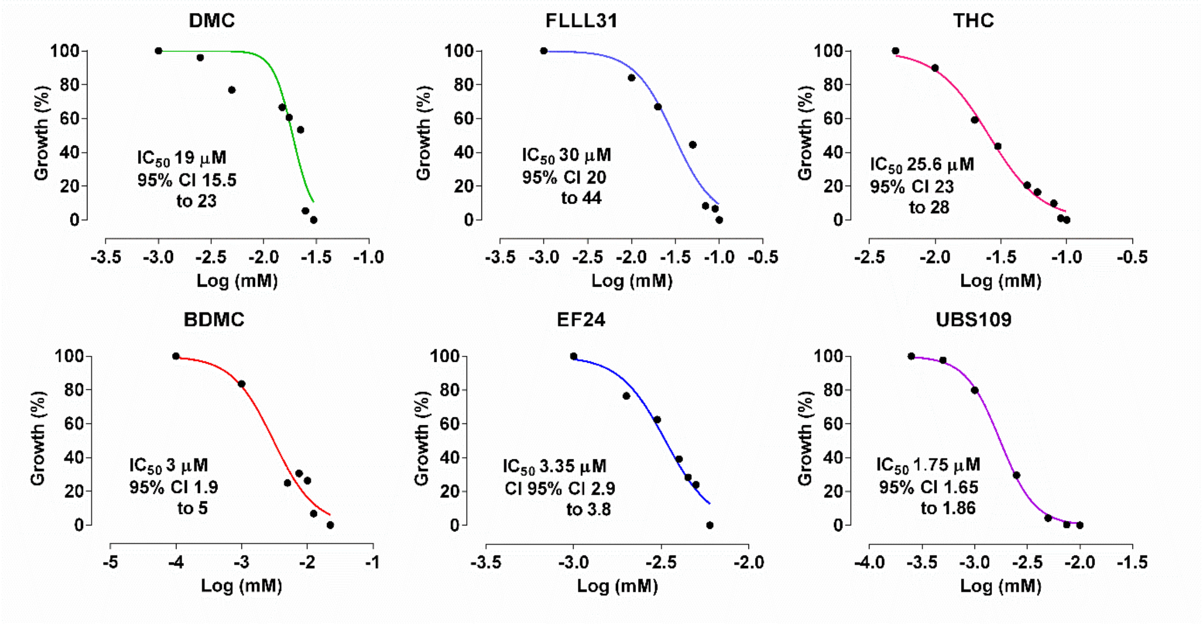

Supplementary Fig. 4. Quantification of the chronic effect of curcumin derivatives on *D. discoideum*. Using a range of structurally related compounds, concentration dependent responses were determined for *D. discoideum* cell growth, and illustrated as the normalised reduction in growth against the Log (concentration) of each compound (shown with errors based on the 95% confidence intervals), enabling calculation of an IC_{50} values and 95% confidence intervals for each compound.

Disease Models & Mechanisms • Supplementary information

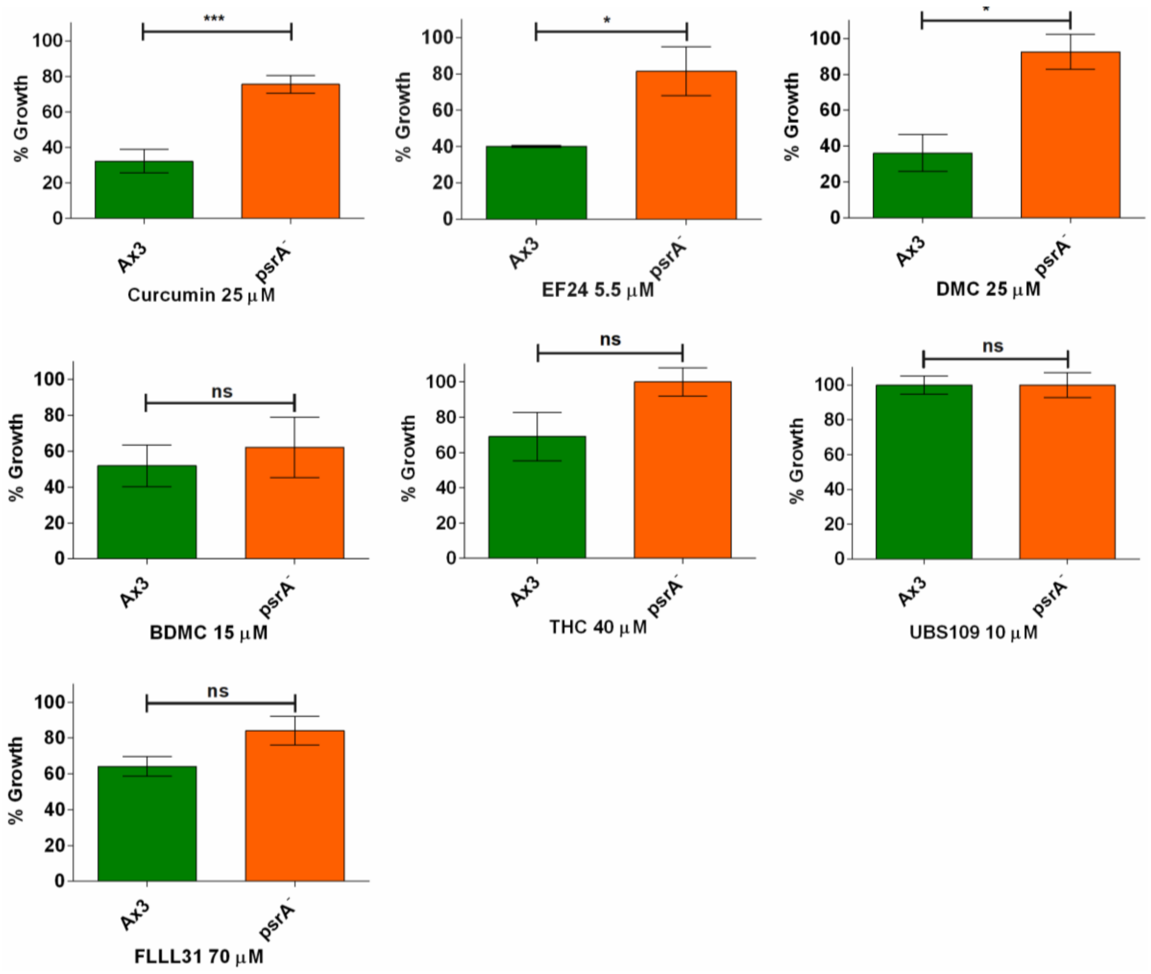

Supplementary Fig. 5. Growth inhibition assay - Ax3 and psrA$^-$ in presence of curcumin and its derivatives. Cells were grown in shaking suspension in presence of different curcumin derivatives. Analysis with Two-tailed t-test showed that psrA- mutants are resistant to curcumin as compared to AX2 (*** $p < 0.001$). psrA- mutants were also resistant to EF24 (* $p < 0.05$), THC (* $p < 0.05$) and DMC (** $p < 0.01$) in comparison to AX2. psrA- mutants were not resistant to BDMC, UBS109 and FLLL31. Data is provided as mean of at least three independent experiments ± SEM.

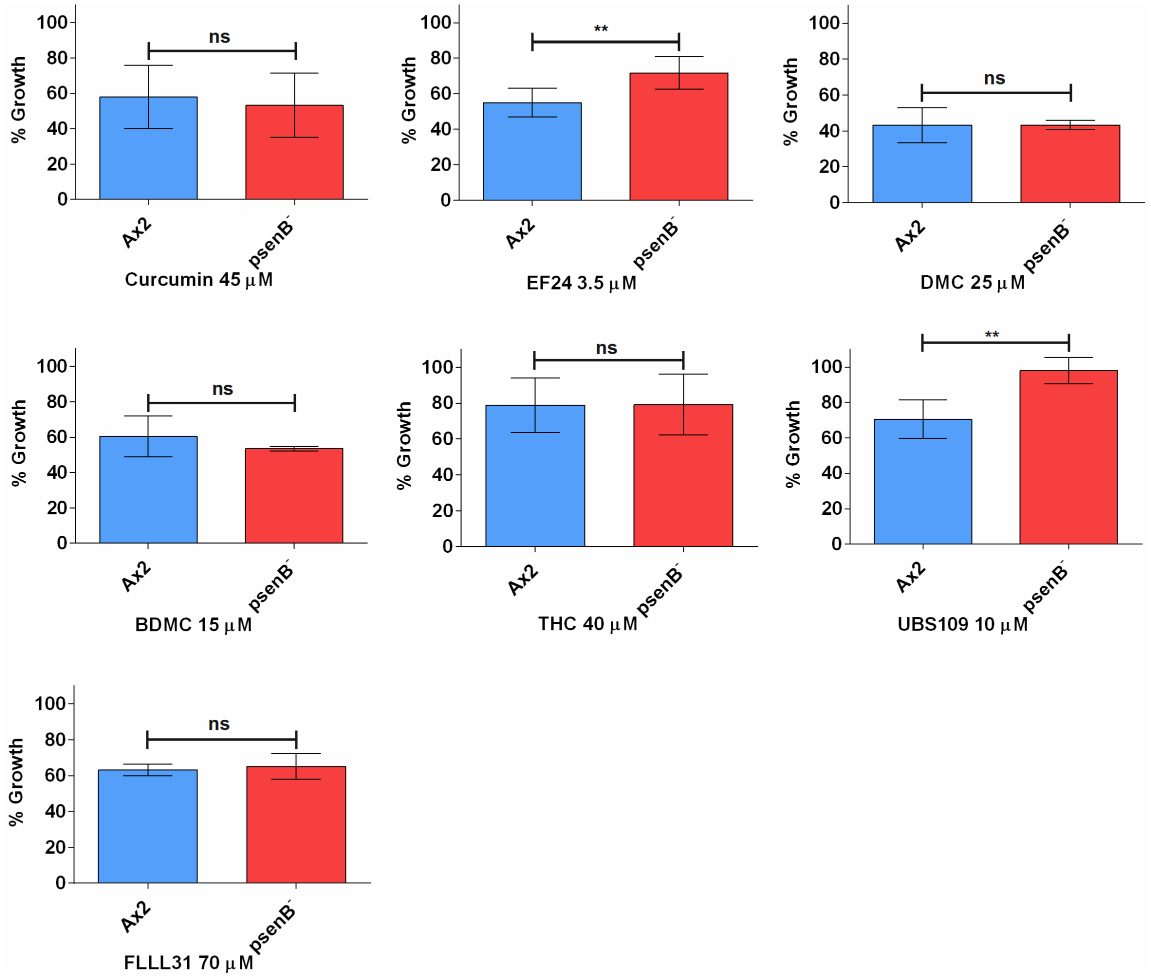

Supplementary Fig. 6. Growth inhibition assay - Ax2 and psenB$^-$ in presence of curcumin and its derivatives. Cells were grown in shaking suspension in presence of different curcumin derivatives. Analysis with Two-tailed t-test showed that psenB- mutants are resistant to EF24 as compared to AX2 (*** $p < 0.001$). Interestingly psenB- mutants were also resistant to UBS109 (** $p < 0.01$) in comparison to AX2. Results showed that the psenB$^-$ mutants were not resistant to curcumin, DMC, BDMC, THC and FLLL31. Data is provided as mean of at least three independent experiments ± SEM

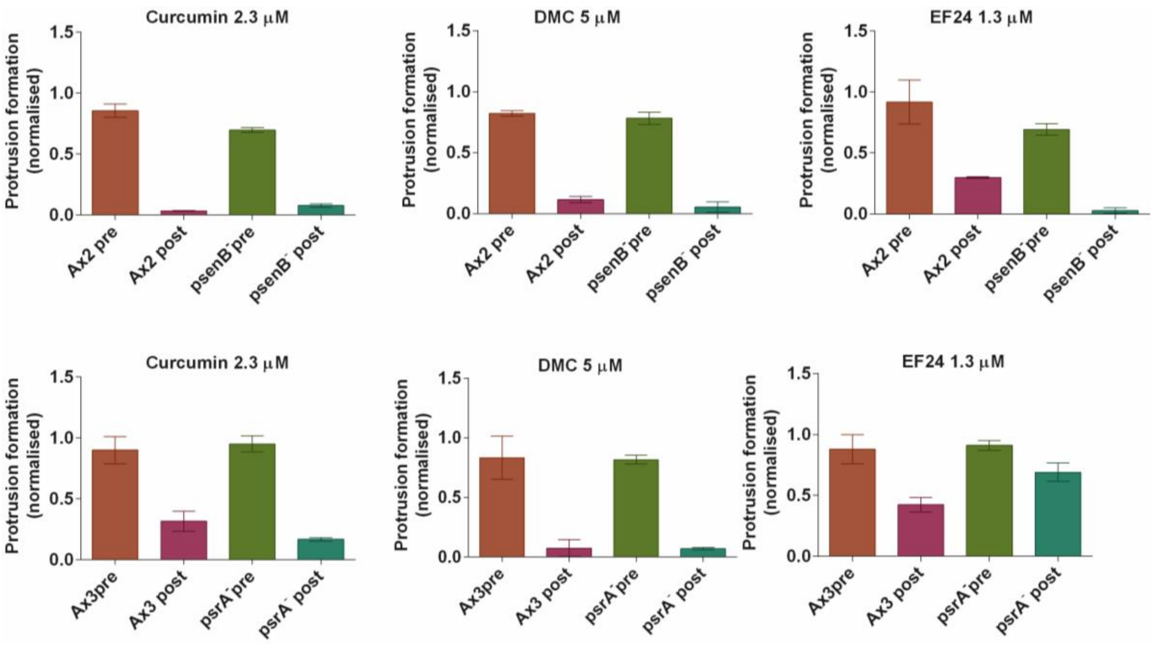

Supplementary Fig. 7. Assessment of the chronic effect of curcumin and its derivatives on *D. discoideum* mutants. psenB and psrA null mutants were exposed to curcumin, DMC and EF24. The mean of the normalised protrusion formation was calculated for the first and the last 5 min for each cell line. The first set of graphs shows that the psenB$^-$ mutant is not resistant to any of the compounds. The second set of graphs illustrates that psrA is sensitive to this range of molecules. Data is provided as mean of at least three independent experiments ± SEM.
